# The complete mitochondrial genome of longlegged spiny lobster *Panulirus longipes* (A. Milne Edwards, 1868)

**DOI:** 10.1080/23802359.2021.1878952

**Published:** 2021-03-01

**Authors:** Hongtao Liu, Liyun Pu

**Affiliations:** a Key Laboratory of Utilization and Conservation for Tropical Marine Bioresources (Hainan Tropical Ocean University), Ministry of Education, Sanya, China; b Hainan Provincial Key Laboratory of Tropical Maricultural Technologies, Hainan Academy of Ocean and Fisheries Sciences, Haikou, China

**Keywords:** *Panulirus longipes*, mitochondrial genome, phylogenetic analysis

## Abstract

In this study, we first determined and characterized the complete mitochondrial genome of longlegged spiny lobster *Panulirus longipes* from South China Sea. The *P. longipes* mitogenome is 15,739 bp long, and consists of 22 tRNA genes, two rRNA genes, 13 protein-coding genes (PCGs), and one control region. The nucleotide composition of *P. longipes* mitogenome is significantly biased (A, G, T, and C was 32.06%, 14.36%, 32.42%, and 21.16%, respectively) with A + T contents of 64.48%. Among 13 PCGs, COX1 gene used an unusual initiation codon CAA, COX1, COX2, ND4 and CYTB genes were ended with an incomplete stop codon T, and ND5 gene with an abnormal stop codon ATT. One microsatellite (C)_10_ was identified in *P. longipes* mitogenome located in the control region. Phylogenetic tree showed that *P. longipes* was first clustered with *Panulirus cygnus*, then together with *P. japonicus* and *P. argus*.

*Panulirus longipes*, commonly known as longlegged spiny lobster, its body and especially the abdomen covered with numerous distinct round white spots, belongs to the family Palinuridae. It is native to the tropical and subtropical Indo-Pacific region, its range extends from Madagascar and the east coast of Africa, Mediterranean, India, to Malaysia, Japan, Taiwan, the Philippines, Indonesia, Papua New Guinea and northern Australia (George and Rao 1965; Pillai and Thirumilu 2007; Spanier and Friedmann 2019). The occurrence of *P. longipes* is usually in clear or slightly turbid water at depths less than about 18 m (59 ft), but exceptionally as deep as 122 m (400 ft) on rocky and coral reefs. They are nocturnal and not gregarious, and feed on mollusks and other bottom-dwelling marine invertebrates. They are caught throughout most of its range and sold fresh and frozen in local markets or as an important export items due to its food values and excellent market demand and price. Till now there are no population figures available but it is likely that it is being overexploitation in parts of its range. Early studies focused on its basic biology such as physiology, development and reproduction, and environmental factors effects. Recently the researchers have paid more attention to its molecular phylogeny. An analysis of partial nucleotides sequences of the mitochondrial cytochrome oxidase subunit I gene(COI) provides molecular evidences supporting the recognition of the morphologically similar species *Panulirus femoristriga*, P*anulirus longipes bispinosus* and *Panulirus longipes longipes* (Juinio-Meñez and Ravago 2002). The phylogenetic relationships of *P. longipes* in Palinuridae were preliminary studied using portions of the large-subunit ribosomal RNA(16S) and COI (Ptacek et al. 2001).

The specimens of *P. longipes* were obtained from Qionghai, China (N19°18′48.37″, E110°40′20.21″), and deposited at the marine crustacean specimen room (Liyun Pu, puliyun@163.com) under the voucher number C20190709PL in Qionghai research base of Hainan Academy of Ocean and Fisheries Sciences. The libraries with an average length of 350 bp were constructed using the NexteraXT DNA Library Preparation Kit, and sequencing was performed on the Illumina Novaseq platform (Total Genomics Solution Limited, SZHT) with the 150 bp average length of the generated reads. The complete mitochondrial genome of *P. longipes* were assembled with 4.64 G clean reads using the *de novo* assembler SPAdes 3.11.0 (Bankevich et al. 2012), and annotated using the MITOS (http://mitos.bioinf.uni-leipzig.de/index.py). The phylogenetic analysis was carried out based on the 13 PCGs encoded by 20 Palinuroidea mitogenomes available in GenBank (Table S2) using IQ-TREE v1.6.12 by maximum likelihood (ML) method with 1000 bootstrap replicates.

The whole mitogenome of *P. longipes* (Table S1) is 15,739 bp in size (GenBank Accession No. MN817128). The base content was 32.06% A, 14.36% G, 32.42% T, and 21.16% C. The 64.48% of (A + T) showed great preference to AT. It consists of 22 tRNA genes, two rRNA genes, 13 protein-coding genes (PCGs), and one control region. Four PCGs (ND1, ND4, ND4L and ND5) and seven tRNA genes were located on the light strand, the others were encoded by the heavy strand.

The 22 tRNA genes in *P. longipes* mitogenome vary in length from 63 bp to 73 bp. tRNA-Leu and tRNA-Ser both have two type copies, respectively. The 12S rRNA is 851 bp and located between tRNA-Val and the control region, and the 16S rRNA is 1365 bp, located between tRNA-Val and tRNA-Leu. Except COX1 using an unusual CAA as the start codon, the others use a normal initiation codon ATN or GTG. Simultaneously, we also found that except for eight PCGs using TAA or TAG, the stop codon of the other five genes were abnormal: COX1, COX2, ND4, and CYTB use a single base T; ND5 uses ATT. The control region is 804 bp, located between 12S rRNA and tRNA-Ile. Interestingly, one microsatellite (SSR) (C)_10_ was identified in the control region in *P. longipes* mitogenome using MISA (Beier et al. 2017), and also found similar in some other closely related species with different number of repetitions (Figure S1).

The phylogenetic tree (Figure 1) showed that *P. longipes* was first clustered with *Panulirus cygnus*, then formed a clade with *P. japonicus* and *P. argus* which further clarified the phylogenetic relationships of the species of *Panulirus* in the family Palinuridae. It was concordant with previous analyses based on mitochondrial cytochrome oxidase I gene (Juinio-Meñez and Ravago 2002; Chow et al. 2006).

**Figure 1.**
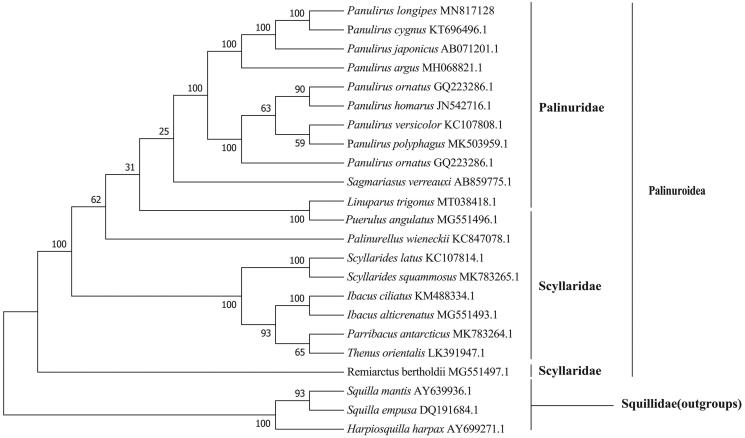
The maximum likelihood tree of *P. longipes* and 23 other species based on 13 PCGs. *Harpiosquilla harpax, Squilla empusa and Squilla mantis* were used as outgroups.

## Data Availability

The genome sequence data that support the findings of this study are openly available in GenBank of NCBI at (https://www.ncbi.nlm.nih.gov/) under the accession no. MN817128. The associated BioProject, SRA, and Bio-Sample numbers are PRJNA679080, SRR13076875, and SAMN16823636, respectively.
